# Determining underlying influences of data variability in the novel object recognition paradigm as used with young pigs

**DOI:** 10.3389/fnbeh.2024.1434489

**Published:** 2024-08-27

**Authors:** Rebecca K. Golden, Ryan N. Dilger

**Affiliations:** ^1^Neuroscience Program, University of Illinois, Urbana, IL, United States; ^2^Division of Nutritional Sciences, University of Illinois, Urbana, IL, United States; ^3^Department of Animal Sciences, University of Illinois, Urbana, IL, United States

**Keywords:** meta-analysis, object recognition, novelty preference, young pigs, cognition

## Abstract

The novel object recognition (NOR) paradigm is a cognitive test that has been used with many species to detect differences in ability. Various iterations of the paradigm have been implemented, making it difficult to compare results both within and across species. Interpretations of the results are equally diverse, threatening the integrity of the paradigm. These inconsistencies have prompted a deeper dive into the variability of the resultant data. For the purposes of this meta-analysis, data originated from 12 studies involving 367 pigs that were subjected to the same NOR paradigm beginning between postnatal days 21 and 24. The main cognitive measure from the NOR paradigm is recognition index (RI), which was the focus of most of the analyses in this meta-analysis. RI was chosen as the main outcome as it determines a pig’s preference for novelty, an innate behavior of cognitively intact pigs. A histogram of RI values (range 0 to 1) showed a bimodal distribution skewed to the right, suggesting that the interpretation of positive performance on the task may need to be stricter. Correlational analyses proved that the number of investigations and investigation time with both the novel and familiar objects were the strongest predictors of resultant RI values. Objective data inclusion criteria were then considered to eliminate non-compliant pigs. Results indicated that requiring at least 5 s of investigation over a minimum of 3 investigations with the novel object reduced overall variability for RI with a concomitant increase in the mean. Further analyses showed that pigs preferred to spend more time with and interact more with the novel object across the entire testing trial, especially in the first minute. Together, these findings suggest that future interpretations of NOR should consider applying stricter statistical analyses as well as additional data processing, such as binning, with emphasis on novel object and familiar object investigation. Overall, modifications to the existing iterations of the NOR paradigm are necessary to improve paradigm reliability.

## 1 Introduction

The novel object recognition (NOR) paradigm is a tool used for investigating cognitive processes and memory in various species, offering insights into the inner workings of the brain. Original development of this paradigm was done with the intent to study human infants (Fantz, 1964) as a means to assess cognition independent of spoken language (Piaget and Inhelder, 1969). The task was then translated to animal models, beginning with rodents in 1988 (Ennaceur and Delacour, 1988). It was later translated for usage with pigs, mainly in the biomedical field (Moustgaard et al., 2002). Usage of the paradigm in animals often revolves around determining the impact of impairment models such as Alzheimer’s disease (AD), acquired hydrocephalus, and ischemic strokes on cognitive abilities (McAllister et al., 2021; Søndergaard et al., 2012; Kaiser et al., 2021) due to the innate preference for novelty in the environment that cognitively intact pigs express (Wood-Gush and Vestergaard, 1991). However, its usage is expanding to investigate the influence of early-life nutrition on brain and cognitive development (Fil et al., 2021a; Fleming et al., 2018; Fang et al., 2020).

Literature, both across and within species, varies greatly in terms of NOR testing procedures and interpretation of results. This precludes direct comparison of results between studies and brings into question the integrity of findings. The base of the paradigm includes three phases: habituation, sample, and test. However, how and when these phases are conducted vary from study to study. Literature focused specifically on use of the NOR paradigm with pigs is not immune to discrepancies in protocol design. While a study utilizing an AD model did not see any performance differences between control pigs and AD pigs (Søndergaard et al., 2012), the induction of a stroke did invoke performance differences from before to after the event (Kaiser et al., 2021). Similar inconsistencies have been observed with dietary supplementation models, with some evidence suggesting enhanced cognitive abilities with supplementation (Fang et al., 2020; Fleming et al., 2019) and others failing to do so (Buddington et al., 2018; Parois et al., 2021). Despite foundational literature agreeing that tasks requiring memory utilize the functionality of the hippocampus (Eichenbaum, 2004; Scoville and Milner, 1957), lack of consistent results from the NOR paradigm have caused some researchers to question this premise (Garcia-Bonilla et al., 2023; Oliveira et al., 2010).

While the NOR paradigm is commonly used with rodents and human infants, the usage with pigs is far less common, limiting the amount of available data for comparison. Additionally, although the pig NOR paradigm design and procedures have been informed by rodent literature, discrepancies still exist due to species differences. As such, comparison between species is difficult. Given the procedural inconsistencies applied to the NOR paradigm in pigs, the objective herein was to systematically review and analyze data in order to elucidate sources of variation. Our approach includes analysis of 12 studies where 367 young domestic pigs were subjected to the NOR paradigm using identical procedures to identify idiosyncratic trends associated with the main cognitive outcome [recognition index (RI)], predictive measures of RI values, data inclusion criteria, and other exploratory measures.

## 2 Materials and methods

### 2.1 Data points included

The NOR paradigm was applied by a single laboratory, the Piglet Nutrition and Cognition Laboratory (PNCL) at the University of Illinois at Urbana-Champaign, in a total of 14 studies with young pigs over the course of 9 years. The studies utilizing this iteration of the paradigm were largely focused on dietary interventions during postnatal development, which consisted of either supplementation of specific nutrients above or nutrient deficiency below standard levels. Of the 14 studies, one did not utilize the standard testing procedures (described below) and one utilized a nutrient deficiency model. Nutrient deficient animals experience differences in both physical growth and cognitive development compared with animals provided nutritionally adequate or supplemented diets (Knight and Dilger, 2018; Tveden-Nyborg et al., 2012). As such, raw data were pooled from the remaining 12 studies (Table 1) as they utilized the same testing protocol (described in Section 2.2) and focused on the supplementation of nutrients for which there are no defined physiological requirements. In other words, tested nutrients in the included studies do not have a definitive required amount for healthy growth and as such, animals receiving supplementation at any level cannot be considered deficient. In cases of repeated exposure to the paradigm, only first-exposure data were utilized if, and only if, the procedure matched that of the other studies. All pigs from each of the 12 studies were included regardless of their assigned treatment as all pigs received nutritionally-adequate diets. As a result, the pooled dataset contains data from 386 unique pigs that were all exposed to the same NOR testing protocol. All pigs were obtained from an Institutional Animal Care and Use Committee (IACUC)-approved swine farm. After pooling the data, a total of 19 pigs from various studies were excluded due to lack of any investigation with either object, which has been determined to be a standard outlier removal criterion (Ennaceur and Delacour, 1988). The resultant dataset included 367 data points for the final data analysis.

**TABLE 1:** Data included in pooled dataset^1^.

Study^2^	Total data points	Data points excluded^3^	Citation
1	5	1	Fleming and Dilger, 2017
2	54	6	Sutkus et al., 2022
3	29	2	Fil et al., 2021a
4	21	1	Joung et al., 2020
5	60	0	Golden et al., 2024
6	70	2	Fleming et al., 2020
7	22	0	Fleming et al., 2019
8	49	6	Fil et al., 2021b
9	18	0	N/P
10	16	0	N/P
11	13	0	N/P
12	29	1	N/P

^1^N/P, not published.

^2^Study numbers have been arbitrarily assigned and do not reflect any information about the data.

^3^Data points excluded due to lack of investigation of either object.

### 2.2 Common testing procedure

All studies included in the pooled dataset followed the same NOR testing procedures, including arena and object designs, which were previously described in detail by Fleming and Dilger (2017). This test is based on the inherent preference for novelty that pigs express (Wood-Gush and Vestergaard, 1991). The original human infant paradigm focused on testing around 3 months of age (Fantz, 1964). In terms of brain development, a one-week-old pig is said to be equivalent to a one-month-old human infant (Conrad and Johnson, 2015). As such, intact male domestic pigs were subjected to the NOR paradigm beginning between postnatal day 21 and 24. All pigs were of similar genetic background, which included Pig Improvement Company (PIC; Hendersonville, TN) Line 3 dams artificially inseminated utilizing a pooled semen source. Prior to testing, all pigs were individually reared at PNCL in cages that allowed pigs to see, hear, and smell, but not directly touch, adjacent pigs. The cages did not include any enrichment devices. Pigs were handled daily by rotating shifts of study personnel to capture body weights, body condition scores, and overall health status of young pigs, per established protocols. NOR testing procedures always started between 0900 and 1000 h, which was after the initial availability of milk replacer each morning. Once pigs were placed in the NOR testing arena, study personnel left the testing room and closed a set of doors to minimize or eliminate distractions for the pigs.

The paradigm consisted of three phases run over the course of five consecutive days. Phase one was habituation, which consisted of placing the pig in an empty testing arena (1.83 m × 1.83 m × 1.16 m; L × W × H; ShapeMaster, Ogden, IL, United States) and allowing exploration for 10 min on two consecutive days. Phase two was the sample day, during which pigs were placed back into the arena, this time containing two identical objects secured to the floor (center-left and center-right). Pigs were allowed to explore for 5 min in this task phase. After a 48-h delay, pigs underwent phase three, the test day. Pigs were returned to the testing arena, now containing one familiar object from the sample phase and one novel object, both secured to the floor, and pigs were once again allowed to explore for 5 min. Objects utilized for the paradigm were designed to eliminate inherent biases such as shape, familiarity, interactivity, and color preference (Fleming and Dilger, 2017). The arena and objects were cleaned with diluted bleach and hot water between pigs to eliminate odors and excrement. Novel and familiar object designations were counterbalanced to mitigate object preference. Similarly, the side of the arena on which the novel object was presented was also counterbalanced to mitigate inherent preferences. All phases were video recorded and later analyzed by trained, unbiased personnel (i.e., blinded to treatment).

Video analysis for all studies was done using EthoVision XT 11 (Noldus Information Technology, Wageningen, The Netherlands), Adobe Premiere [Adobe Inc. (1999–2019). *Adobe Premiere Pro* (v14.0) (Software). San Jose, CA: Adobe Inc.], or Loopy (Loopbio GmbH, Vienna, Austria).^1^ Video was recorded at a rate of 30 fps and analysis software allowed for frame-by-frame detailing. The trial started immediately after placing the pig in the arena and no researchers were visible within the camera scope. Trial conclusion was determined by the opening of the arena after 5 min. Behavior analysis criteria for all studies was based on Fleming and Dilger (2017). Given that pigs interact with surroundings using their snout, investigations were determined based on rooting-like behaviors. Investigation intention was determined by the snout of the pig being pointed toward an object with subsequent frames indicating fulfillment of the investigation. The investigation event began when the snout was oriented properly and approximately 10 cm away from the object. The investigation event ended when the pig turned its head so the snout was no longer oriented toward the object. One “beginning” marker and the immediate next “end” marker dictated a single investigation event and the change in time between those markers was used to determine the duration of the event. Raw data was then exported from the analysis software before being processed into the measures described below.

The main cognitive outcome from the NOR paradigm is RI, which is defined as the amount of time spent interacting with the novel object as a proportion of total time spent interacting with both objects. RI values may also be compared to the chance value (0.50) via a t-test. The chance level is set at 0.50 for this rendition of the paradigm given that pigs have only two objects to explore. In addition to RI, further exploratory behaviors were quantified including latency to first and last investigation and frequency and duration of investigations. These measures were quantified individually for both the familiar and novel objects, as well as combined for total exploratory behavior measures. Published data from the studies included in the pooled dataset performed outlier removal per each exploratory behavior after the first round of processing. Outliers were determined as having a studentized residual with an absolute value of 3 or greater. In addition, a pre-processing step of removing pigs that engaged in less than 2 s of investigation time with either the novel or familiar objects was applied. This was done with the intent to remove any non-compliant pigs that would negate novelty preference (Fleming and Dilger, 2017).

### 2.3 Statistical analyses

All analyses performed in this meta-analysis were conducted in SAS (RRID:SCR_008567; version 9.3; SAS Inst. Inc., Cary, NC, United States) and Excel [Microsoft Corporation. (2016). Microsoft Excel (Version 2016) (Software). Redmond, WA: Microsoft Corporation]. RI values were calculated as the amount of time spent investigating the novel object over the total investigation time for both objects. Subsequently, RI values were binned in increments of 0.05 (i.e., 0.00–0.049, 0.05–0.099, etc.) to generate a histogram. In conjunction with the histogram, the standard deviation (SD) and coefficient of variation (CV) were also calculated. The RI values were then compared to the rodent discrimination index (DI), which was calculated as the amount of time spent exploring the novel object minus the time spent exploring the familiar object divided by the total investigation time.

With the understanding that some of the exploratory behaviors directly influence RI values (i.e., RI = novel object investigation time/total investigation time), for the purposes of determining significance, alpha was set at 0.01. A correlation heat map for all NOR outcomes as well as body weight (BW) was generated using Pearson correlation coefficients. Subsequent scatterplots were generated, with individual exploratory behaviors acting as the independent variable and RI as the dependent variable. Cook’s D was used to ascertain whether any individual value was significantly impactful on resultant correlations. A Box-Cox transformation was then applied to 4 of the exploratory behaviors in an attempt to normalize the data. For all descriptive statistical analyses, RI served as the dependent variable. Both forward and backward regression analyses were conducted. Initially, both regression models included the “overall total” exploratory behavior variables. However, the “total” variables were ultimately removed to elucidate which individual object investigations were driving the significance of the total. Continued analysis of the best predictive model for RI was conducted using an R-squared goodness of fit forward selection regression analysis with both Akaike Information Criterion (AIC) and Bayesian Information Criterion (BIC) measures being evaluated. Varying numbers of investigation time and number of investigations were then utilized to assess data inclusion criteria. Mean RI values were recorded after the application of the criteria. Chosen-model data were then binned by minute (i.e., 0–0:59, 1:00–1:59, etc.) per pig and averaged across all studies. Mean investigation time and number of investigations were calculated per object within each bin. A subsequent t-test was used to determine differences in exploratory behaviors per bin, as well as across the whole trial. Variance measures per bin, exploratory behavior, and object were also determined. Mean data from each bin was progressively averaged (i.e., first minute, first 2 min, etc.) and the conjugate standard error (SE) for each exploratory behavior was calculated. SE was calculated as SD divided by the square root of the number of data points.

## 3 Results and discussion

Although rodent literature has informed NOR paradigm procedures for use with pigs, inconsistencies in paradigm procedures both within and across species inhibit comparisons between studies, calling into question the robustness and integrity of the paradigm. Despite the abundant use of the NOR paradigm with rodents, species differences make it difficult to compare and interpret findings. In addition, due to the specialized area of research in which the pig iteration of the NOR paradigm is utilized, there is a lack of species-specific information and data that is available. As such, this meta-analysis aims to determine sources of variation in data via in-depth analyses of data from studies utilizing the same NOR paradigm, specifically in young pigs.

### 3.1 What are the RI distribution trends?

Theoretically, RI is an indicator of cognitive ability as the pig must be capable of identifying which object is familiar to preferentially interact with the novel object. As such, dissection of the dataset began by looking at the distribution and trends for this outcome to delineate underlying influences of variation in the resultant data.

#### 3.1.1 Overall RI trends

**FIGURE 1:**
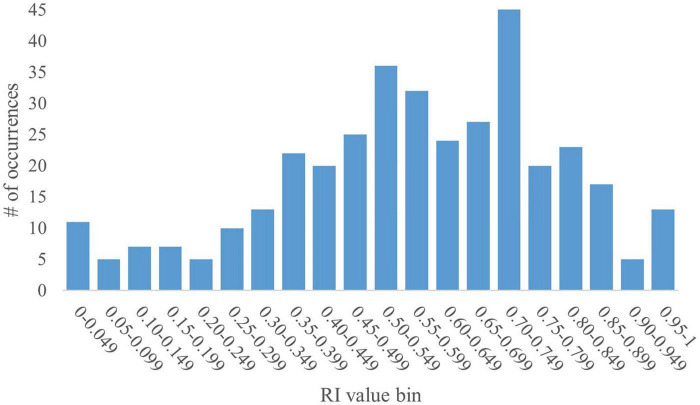
Full histogram of RI values in young pigs. A histogram of the distribution of all RI values (range: 0–1) from the pooled dataset in bins of 0.05 (i.e., 0.00–0.049, 0.05–0.099, etc.) was generated for the full dataset of 367 pigs subjected to a common NOR paradigm. The y-axis represents the number of data points from the pooled dataset that fall within a specific bin. The histogram shows a bimodal distribution skewed to the right with peaks at the 0.50–0.549 and 0.70–0.749 bins. The data produce a mean RI value of 0.57, with an SD of 0.23 and a CV of 40%.

The pooled dataset contained 367 data points. Given that RI is a ratio, values can range from 0 to 1. As such, the dataset yielded a mean RI value of 0.57 with a standard deviation (SD) of 0.23. A histogram generated using RI values in increments of 0.05 revealed a bimodal distribution skewed to the right, with peaks within the 0.50–0.549 and the 0.70–0.749 bins (Figure 1). The bin value of 0.05 allowed for granular analyses, including performance trends and determination of outstanding RI values. The SD for any given bin is small ( ≤ 0.04). However, overall, the data present high variability, as supported by a coefficient of variation (CV) of 40%. That said, except for the first two bins (0.00–0.049 and 0.05–0.099; CV = 116% and 34%, respectively), the CV for any given bin was relatively low ( ≤ 5%).

The interpretation of RI values is based on the chance level, which is 0.50 in the case of this NOR testing protocol, given the 2 objects to explore. As such, current interpretation of RI values states that any pig that produces an RI value above that of chance accurately identified the novel object and therefore exhibits recognition of the familiar object. This presents the problem of RI evaluation being binary: either the pig does exhibit recognition memory, or it does not. However, it does not distinguish between two pigs that produce RI values above or below that of chance. For example, a pig that produces an RI value of 0.52 is interpreted no differently than a pig that produces an RI value of 0.83. The same is true for pigs producing an RI value below that of chance. The use of a t-test to evaluate statistical difference from the chance value further complicates the interpretation of RI values. While a pig that produces an RI value of 0.79 may be statistically above chance and a pig producing an RI value of 0.54 may not be statistically above chance, biologically, it cannot be said that the former pig learned “better” or “more” than the latter. Moreover, such statistical tests are completely dependent on the within-study variability estimate and do not enable a global interpretive view.

With the current interpretation standards, preference for novelty has been established as any value above 0.50, while preference for familiarity is any value at or below 0.50. Using this standard, the current dataset results in 121 pigs (33%) showing preference toward the familiar object while 246 (67%) show preference toward the novel object. Literature detailing inherent pig behaviors suggests that all healthy, cognitively intact pigs would express novelty preference (Wood-Gush and Vestergaard, 1991). However, despite attempts to mitigate object biases and distractions within the testing arena, some pigs may express preference for a specific object rather than seeking novelty, thereby leading to individual variability that is not captured or interpreted in the current NOR paradigm for pigs.

Rodent literature often utilizes DI to measure object recognition, as opposed to RI (Ennaceur and Delacour, 1988). The calculation for DI results in a scale from −1 to 1, where a negative DI value corresponds to more time spent with the familiar object, a positive value corresponds to more time spent with the novel object, and a value of zero corresponds to having no preference. Relating this to RI interpretation, a negative DI value is synonymous with an RI value at or below 0.50 and a positive DI value is synonymous with an RI value greater than 0.50. For reference, a DI value of −0.5 is equal to an RI of 0.25, DI = 0.0 is equivalent to RI = 0.50, and DI = 0.5 is equivalent to RI = 0.75. The current interpretation of RI values does not have an immediately identifiable range to indicate pigs that expressed no object preference (Table 2). Instead, determining null preference requires a subsequent t-test to determine significance from that of chance. Applying the DI calculation to the current dataset resulted in a mean DI of 0.14 (RI ≈ 0.57) with an SD of 0.46 and CV of 321% (data not shown). As with RI, the DI values from this dataset vary greatly. Although the distributions of object preference as determined by RI and DI are similar, the switch to DI may be warranted as it provides an immediately identifiable null category for object preference. That said, with current statistical analyses, high replication would still be necessary to produce a statistically significant mean RI value, due to high variability.

**TABLE 2:** RI versus DI object preference comparison^1^.

Object preference	RI	DI
Preference for familiar	121 (33%)	123 (34%)
No preference	–	3 (< 1%)
Preference for novel	246 (67%)	241 (66%)

^1^The percentage in parentheses listed with each count is the percentage of the dataset to which the criterion applies. DI, discrimination index; RI, recognition index.

The NOR paradigm, and subsequent DI calculation, for rodents is mainly used to determine cognitive differences between control animals and those with some type of brain impairment. The result is a clear distinction in cognitive abilities when comparing DI values between the two groups (Aggleton et al., 2010; Aubele et al., 2009; Burke et al., 2010). However, research applying NOR utilizing various breeds of pigs as brain impairment models (e.g., AD or acquired hydrocephalus) has not been able to replicate the same distinction between control and treatment groups as in rodents (Garcia-Bonilla et al., 2023; McAllister et al., 2021; Søndergaard et al., 2012). Given that the pooled dataset utilized herein derives from dietary supplementation models, it may be possible that the distinction in cognitive abilities between groups is not great enough or that NOR is not sensitive enough to detect differences when all pigs are healthy, developmentally ‘typical’, and receiving nutritionally adequate diets.

#### 3.1.2 Trends for RI value bin 0.70–0.749

**FIGURE 2:**
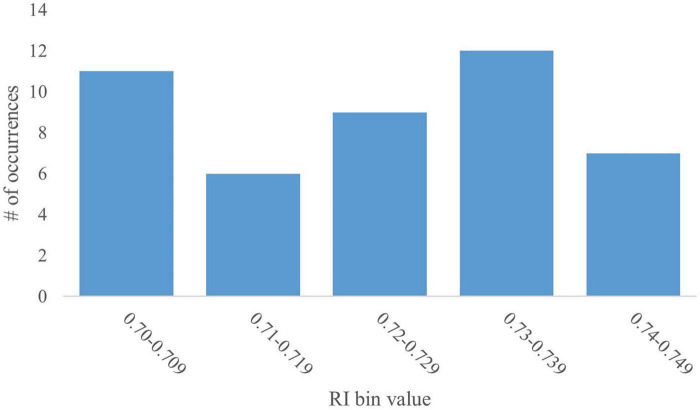
Distribution of the 0.70–0.749 RI value bin. A histogram of the distribution of the RI values that fall within the 0.70–0.749 bin were further analyzed in increments of 0.01 (i.e., 0.70–0.709, 0.71–0.719, etc.). The histogram shows no trend in distribution and low variability within the data subset.

Given the lack of normality in the overarching RI histogram, further analyses of the peak at the 0.70–0.749 bin were conducted. The peak at 0.70–0.749 contains 45 data-points (12% of the overall dataset). The bin was further broken down into sub-bins of 0.01. A subsequent histogram was generated to deduce data distribution (Figure 2). The mean RI value was 0.73 with an SD of 0.014 and a CV of 1.9%. This relatively low variability, as apparent by the SD and CV, is to be expected given the examination of a subsection of data.

Correlational analyses were conducted between the RI values in this bin as well as the paired exploratory behaviors to determine which variables may be influencing this peak. Results indicated that none of the exploratory behaviors of the objects, either independently or in combination, correlated with the RI values in this bin. Rodent literature suggests that animals with no intervention preferentially spend time with the novel object (Silvers et al., 2007). Similar ideas have been proposed for pig, as well (Wood-Gush and Vestergaard, 1991). A possible interpretation of the two peaks may be to categorize the pigs that produced RI values in the 0.50–0.549 bin as having expressed ‘random choice’ while those that produced RI values in the 0.70–0.749 bin expressed ‘good’ or ‘positive’ recognition. However, there is no definitive proof to support that claim. Additional studies would be necessary to determine the validity of that interpretation. As such, a clear explanation for the presence of this peak remains elusive.

### 3.2 Which exploratory behaviors predict RI values?

After analyzing the distribution trends for RI values, focus was shifted to determining which of the exploratory measures most strongly predict RI outcomes. As such, descriptive statistical analyses were conducted utilizing RI as the dependent variable and the exploratory behaviors as the independent variables. As mentioned above, exploratory measures are calculated both independently for each object and combining behaviors for both objects (Table 3).

**TABLE 3:** Abbreviations for exploratory behaviors associated with the NOR paradigm.

Outcome	Novel object (nov)	Familiar object (fam)	Overall total (t)
Number of investigations (n)	nov_n	fam_n	t_n
Total investigation time (inv)	nov_inv	fam_inv	t_inv
Mean investigation time (me)	nov_me	fam_me	t_me
Latency to first investigation (lf)	nov_lf	fam_lf	t_lf
Latency to last investigation (ll)	nov_ll	fam_ll	t_ll

#### 3.2.1 Correlation matrix of NOR outcomes

**FIGURE 3:**
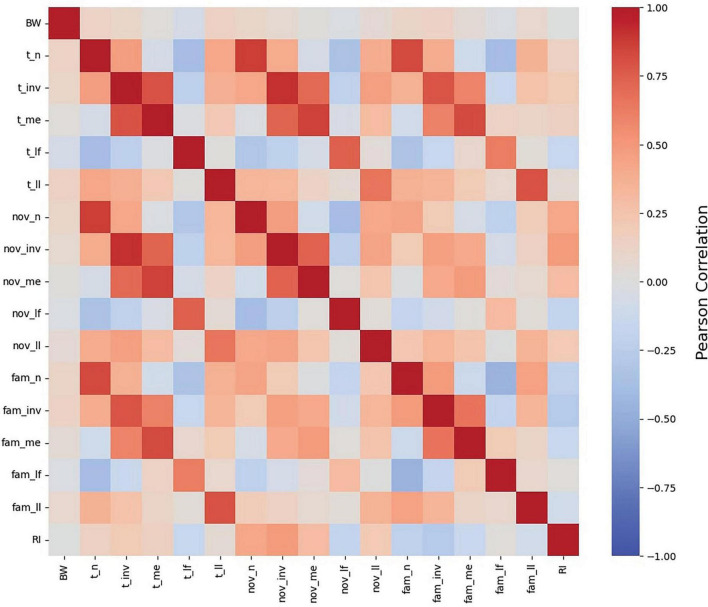
Pearson correlation heat map of NOR measures. A heat map of the relationships between all NOR outcomes, as well as body weight (BW), as determined by Pearson correlation. BW was not correlated to any NOR measure. RI was observed to exhibit strong relationships, both positive and negative, with each exploratory measure excluding latency to first and last investigation with the familiar object as well as latency to the overall last investigation. Abbreviations: BW, body weight; fam, familiar object; nov, novel object; t, both objects; n, number of investigations; inv, investigation time; me, mean investigation time per single investigation; lf, latency to first investigation; ll, latency to last investigation; RI, recognition index.

Initial understanding of variables that influence RI began with a simple correlation matrix (Figure 3). BW was included to assess its association with RI and other exploratory behavior outcomes. Results of the correlation matrix indicate that BW does not correlate to RI (*p* > 0.01). The lack of influence of BW on RI values is supported by the biological fact that the brain grows proportionally to the body (Golden et al., 2023) and by evidence suggesting that intelligence in pigs is only correlated to BW when the brain size relative to BW ratio differs from that of the species’ norm (Minervini et al., 2016). The mean BW of pigs in all studies included in the pooled dataset were well within the typical range for artificially-reared pigs (Vu et al., 2021) and there appear to be no differences in brain development due to rearing style (i.e., sow reared vs. artificially reared pigs) (Fil et al., 2021b).

All exploratory behaviors listed in Table 3 were related to RI (*p* < 0.01) except for t_ll, fam_lf, and fam_ll. Rather than indicative of cognitive ability, latency to interact with novelty may be an indicator of individual pig’s personality (Hessing et al., 1993). Accordingly, pigs that are quick to approach novelty are quick to lose interest and vice versa. However, these authors also indicate that there are pigs that fall in between these two extremes, taking a moderate amount of time to approach novelty and spending a moderate amount of time interacting with the novel object. As such, averaging RI data within a treatment group likely nullifies either extreme case, thereby eliminating the potential association between RI and latency to approach. The remaining exploratory behaviors were related to RI (*p* < 0.01). However, given the interconnectedness of the variables, it is unclear from the heat map which variables are strong predictors of RI. As such, scatterplots of direct exploratory behaviors (i.e., number of investigations and investigation time) were generated to better elucidate the correlations between the variables.

#### 3.2.2 Scatterplots of significant correlations

**FIGURE 4:**
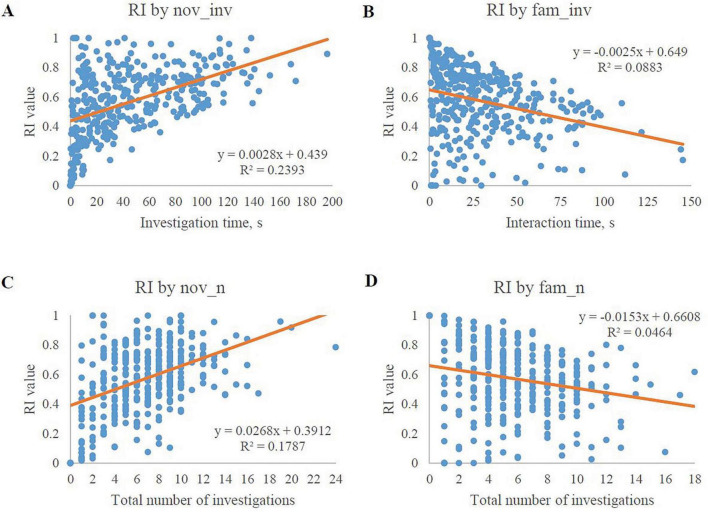
Scatterplots of the best-fit model exploratory behaviors in relation to RI values. Only the 4 most significantly-related exploratory behaviors are plotted. **(A)** The amount of investigation time with the novel object plotted with its corresponding RI value. The graph shows a positive relationship between novel object investigation time and its corresponding RI value. **(B)** The amount of investigation time with the familiar object plotted with its corresponding RI value. The graph shows a negative relationship between familiar object investigation time and its corresponding RI value. **(C)** The number of investigations with the novel object plotted with its corresponding RI value. The graph shows a positive relationship between the number of novel object investigations and its corresponding RI value. **(D)** The number of investigations with the familiar object plotted with its corresponding RI value. The graph shows a negative relationship between the number of familiar object investigations and its corresponding RI value.

Scatterplots were generated utilizing behavioral measures as the independent variable and RI values as the dependent variable to understand associations. Simple regression analyses for the 4 behavioral measures that were most significant are displayed in Figure 4. The scatterplots corroborate the correlations indicated in the heat map with the novel object variables being positively correlated to RI and the familiar object variables being negatively correlated to RI, as supported by the regression parameter estimates.

Given the relationship between RI and the novel object investigations (i.e., the RI value ratio contains nov_inv), the positive relationship between these two variables was expected. The trend line equations indicate that there is greater variability in the novel object investigation behavior data than the familiar object investigation behavior data. However, evaluation of influential data-points via Cook’s D indicated that no individual value(s) significantly impacted the correlations of any of the four exploratory behaviors with RI (data not shown). Furthermore, a Box-Cox transformation was unable to normalize the data by utilizing a selected lambda of 2.5 in order to achieve a confidence interval of 95%. This transformation produced an R-squared value of 0.64 (data not shown). These results may tie back to the ideas presented by Hessing et al. (1993) who suggested that pigs have varying reactions to novelty in their environment and as a result, varying levels of investigation with the novelty. Other studies have also shown similar trends of less variability among familiar object investigation behavior data (Gifford et al., 2007; Golden et al., 2024; Sutkus et al., 2022).

#### 3.2.3 Regression models and model selection of NOR outcomes

To better understand the influence of exploratory behaviors during the NOR paradigm on the subsequent RI values, both forward and backward regression strategies were conducted. Both final models contained the same 8 variables: nov_n, nov_inv, nov_me, nov_lf, nov_ll, fam_n, fam_inv, and fam_me. Similarly, both regression models excluded fam_lf and fam_ll. Exclusion of familiar object latency measures from the regression models mimicked the correlation matrix in which the same latency measures were not observed to be related with RI. As with the correlation matrix, the lack of significance of the familiar object latency measures may be linked back to the findings of Hessing et al. (1993).

The best-fit single-variable model included nov_inv and had an R-squared of 0.224, an AIC of −895.1, and a BIC of −896.4. Given that RI is directly influenced by nov_inv (i.e., RI = nov_inv/t_inv), this finding is expected. The optimal two-variable model contained nov_inv and fam_inv with an R-squared of 0.595, an AIC of −1064.8, and a BIC of −1064.5. After the first two models, the R-squared, AIC, and BIC values begin to plateau. Initially, the best-fit four-variable model contained nov_n, nov_inv, nov_me, and fam_inv and had an R-squared value of 0.654, an AIC of −1102.1, and a BIC of −1101.6. However, the decision to move to the second best-fit four-variable model was made as nov_me is a quotient of nov_inv and nov_n. As such, the second best-fit four-variable model included nov_n, nov_inv, fam_n, and fam_inv and had an R-squared of 0.651, an AIC of −1099.6, and a BIC of −1099.2. This model was ultimately chosen as the best predictive model for RI values. Previous work by Gifford (2005) hypothesized that pigs that spent little to no time investigating the familiar objects during the sample phase would ultimately produce low or neutral RI values due to lack of exposure. However, this was proven to be incorrect, with the author observing no correlation between the measures. Although this meta-analysis focuses strictly on the test phase, it is still of note that correlation analyses found an inverse relationship between familiar object investigative behaviors and RI, and regression analyses confirmed the strong impact of these correlations, which, all together, appears to contradict the work of Gifford (2005). That said, further efforts are warranted to confirm the lack of correlation between sample phase behavior and test phase outcomes.

### 3.3 What should the data inclusion criteria be?

The left and right tails of the RI distribution histogram may indicate non-compliant pigs with a strong preference for either the familiar object (left tail) or novel object (right tail). Applying the previously used investigation criteria to the current dataset resulted in 328 data points with a mean RI of 0.58, an SD of 0.20 and a CV of 34%. These numbers do not differ greatly from the full dataset and still result in high variability. While the 2 s investigation time requirement was based on previous rodent work (Reger et al., 2009), it may not be the best determinant of compliance. Literature does not align with the best practice for determining non-compliance. Some studies have based compliance criteria on sample phase behavior (Bilsland et al., 2008; Broadbent et al., 2010) while others have utilized alternative methods for determining behavioral outliers (Clarke et al., 2010; Reger et al., 2009). As such, utilizing the best-fit model enabled the testing of various non-compliance criteria for the NOR test day.

#### 3.3.1 Investigation time

In keeping with previous methods of determining non-compliance where pigs were required to have a certain amount of investigation time, various investigation time cutoffs were applied to the nov_inv and fam_inv exploratory behaviors and the subsequent mean RI values were recorded. The tested criterion was first applied to the individual measures and then again once measures were combined (Table 4). Results indicated an inverse relationship between fam_inv and nov_inv in relation to RI. As the mean RI value increased with a stricter nov_inv criterion, the mean RI value decreased with the application of the stricter criterion to fam_inv. As such, combining both criteria nullifies the effects and does not yield a mean RI value largely different from the full dataset (0.57). From this table, the most applicable criterion that maximizes the RI value and maintains 88% of the dataset is the requirement of 5 s of investigation with only the novel object, which also reduces variability in the dataset (CV = 31%).

**TABLE 4:** Mean RI value based on required investigation duration criterion^1^.

Duration, s	Only fam_inv	Only nov_inv	Both
x ≥ 2^2^	0.56 (38%)	0.60 (34%)	0.58 (34%)
x ≥ 5	0.56 (37%)	0.61 (31%)	0.59 (30%)
x ≥ 10	0.54 (36%)	0.63 (28%)	0.59 (27%)
x ≥ 15	0.54 (36%)	0.63 (27%)	0.59 (25%)
x ≥ 20	0.53 (35%)	0.64 (27%)	0.58 (25%)

^1^The number in parentheses listed with each mean RI is the coefficient of variation of the dataset after application of exclusionary criteria. Abbreviations: fam_inv, total investigation time with the familiar object; nov_inv, total investigation time with the novel object.

^2^Previously applied criterion; listed for reference.

Previous studies involving pigs and rodents have utilized investigation time as a compliance criterion, removing animals that did not meet a defined amount of time. While vague in a specific amount of time, some studies have reported removing animals that expressed “low levels” of investigation time (Bilsland et al., 2008; Ennaceur and Delacour, 1988). Other studies have analyzed data strictly from animals that successfully “learned” the task, investigating the novel object significantly more than the familiar (Clarke et al., 2010). Another rodent study utilized a two-step inclusion criteria process, requiring at least 5 s of investigation with at least 1 s of investigation of each object (Anderson et al., 2004). As such, the application of 5 s of investigation is supported by previous research and beneficial to reducing data variability.

#### 3.3.2 Number of investigations

Although not commonly used to determine compliant animals, the impact of number of required investigations on the resultant RI value was evaluated. The mean RI value and coefficient of variation after the application of the criterion are listed in Table 5. Results indicated an inverse relationship between investigations with the familiar object and investigations with the novel object in relation to RI, similar to the trend observed with investigation time. As the required number of investigations with the familiar object increased, the mean RI value decreased. The opposite occurred for the number of investigations with the novel object, where the mean RI value was directly proportional to the required number of investigations. As such, the most applicable criterion that maximizes the RI value and maintains 88% of the dataset is the requirement for pigs to have at least 3 investigations with the novel object, which also decreases data variability (CV = 32%).

**TABLE 5:** Mean RI value based on required number of investigations criterion^1^.

Number of investigations	Only fam_n	Only nov_n	Both
x ≥ 1	0.56 (40%)	0.58 (37%)	0.57 (37%)
x ≥ 2	0.57 (38%)	0.60 (34%)	0.58 (33%)
x ≥ 3	0.56 (37%)	0.61 (32%)	0.59 (31%)
x ≥ 4	0.56 (36%)	0.61 (31%)	0.59 (30%)
x ≥ 5	0.55 (36%)	0.62 (29%)	0.58 (29%)

^1^The number in parentheses listed with each mean RI is the coefficient of variation of the dataset after application of exclusionary criteria Abbreviations: fam_n, total number of investigations with the familiar object; nov_n, total number of investigations with the novel object.

Although not specifically stated in terms of number of investigations, previous literature requiring a set amount of interaction time with both objects indicates a requirement of at least one interaction with both objects. For example, Reger et al. (2009) required at least 2 s of investigation of both objects. This criterion leads to ensuring that the animal has also investigated each object at least once. Anderson et al. (2004) maintained a similar inclusion criterion, also dictated in terms of amount of investigation time as opposed to number of interactions. Requiring at least 1 s of investigation with each object once again ensures that the animal has investigated both the novel and familiar objects.

**FIGURE 5:**
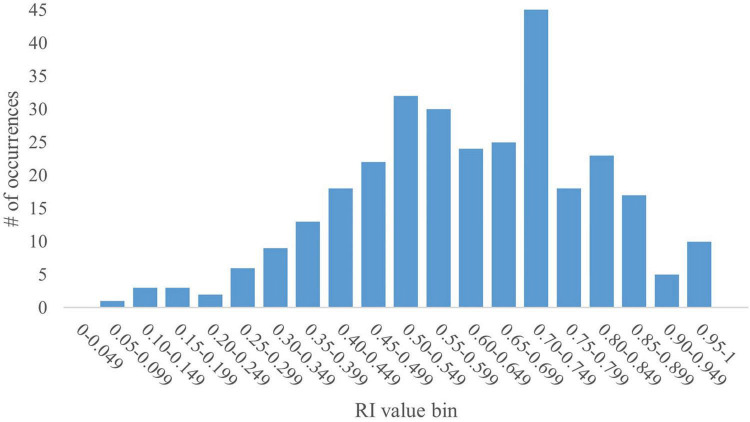
Histogram of RI values of young pigs that meet compliance criteria. A histogram of the distribution of RI values in bins of 0.05 (i.e., 0.00–0.049, 0.05–0.099, etc.) that meet the new data inclusion criteria (5 sec of investigation over 3 investigations) was generated. The new dataset contained 306 data points, which is 83% of the full dataset. The histogram shows a bimodal distribution skewed to the right with peaks at the 0.50–0.549 and 0.70–0.749 bins. The data produced a mean RI value of 0.64, with an SD of 0.18 and a CV of 30%.

The application of the newly determined criteria, at least 5 s of investigation over at least 3 investigations with the novel object, results in the current dataset retaining 306 data points (83% of the total dataset) with a mean RI value of 0.62, an SD of 0.18, and a CV of 30%. The resultant dataset decreases the variability of the data from the whole dataset as well as the original compliance criteria, subsequently increasing the mean RI value. Despite this, the data still produce a bimodal distribution skewed to the right (Figure 5). Nonetheless, these data inclusion criteria applied to an independent NOR study may help to increase the paradigm’s sensitivity to minute cognitive differences.

### 3.4 How do the exploratory behaviors change across the test trial?

Examination of the exploratory behaviors was conducted to elucidate trends and further understand the underlying variability of NOR outcomes. Specifically, the chosen model variable trends have been examined by means of trial binning.

#### 3.4.1 Binning by time across the testing period

**FIGURE 6:**
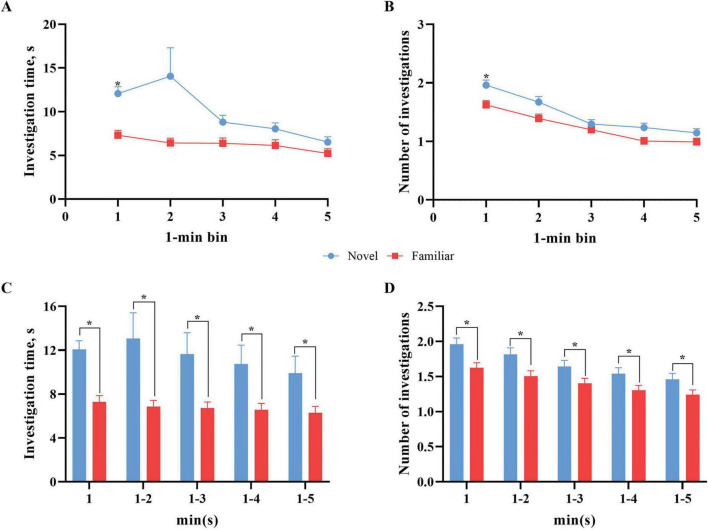
Novel object versus familiar object exploratory behavior per 1-min bins. Error bars represent the standard error of the mean. **(A)** The mean investigation time per 1-min bin across the 5-min test phase. Overall, there was numerically more investigation with the novel object across all duration bins. The mean investigation time (s) with the novel object differed (*p* < 0.01) from the mean investigation time (s) with the familiar object in the first bin (0–0:59). **(B)** The total number of investigations per 1-min bin across the 5-min test phase. Error bars for both objects are shown but some are too small to visualize. Overall, there were numerically more investigations with the novel object across all bins. The total number of investigations with the novel object was greater (*p* < 0.01) than that for the familiar object in the first bin (0–0:59). **(C)** The mean investigation time (s) as the test trial progresses (i.e., first minute, first 2 min, first three minutes, etc.) per object. Pigs continued to investigate the novel object significantly more (*p* < 0.01) than the familiar object for the entire progression of the test trial. **(D)** The mean number of interactions as the test trial progresses (i.e., first minute, first 2 min, first 3 min, etc.) per object. Pigs investigated the novel object significantly more (*p* < 0.01) than the familiar object for the entire progression of the test trial. *Means differ (*P* < 0.05).

Exploratory behavior data from the chosen model were also examined independently of RI by applying the data pre-processing technique of binning to reduce the effects of minor observation errors over the time-dependent course of an NOR test for individual pigs. Published data were reported as results from the whole test trial that was conducted over a 5-min period. Binning was conducted to determine exploratory trends that account for time-dependent changes in behavior, including waning novelty preference, attention, focus, etc. Due to unavailable data, the resultant pooled dataset, strictly for binning purposes, consisted of 354 independent subjects across 10 studies. Bin 2 data for both the novel object and the familiar were also unavailable for 11 pigs, but data from the remaining bins for these pigs were still utilized in analyses. Figure 6 displays the comparison of exploratory behaviors with the novel and familiar object data per bin. Specifically, the mean amount of investigation time per bin and the number of investigations per bin were compared.

Our data with young pigs indicate more investigation of the novel object across time. Specifically of note is the spike in investigation time in bin 2 for the novel object before decreasing. This pattern of investigation of the novel object is concurrent with previous studies that found the same pattern of an initial increase to a peak early in the trial before decreased investigation in the remaining time (Gifford et al., 2007; Wood-Gush and Vestergaard, 1991). Despite the novel object investigation time peak within bin 2, investigation behaviors only differed (*p* < 0.01) by object type in bin 1 due to greater variability in bin 2. Variance for both novel and familiar object investigation time ranged from 103.5 s to 224.6 s, except for novel object bin 2 which had a variance of 3,629.9 s. Similar trends for significant differences between and variance in investigation of the objects were observed for the number of investigations per bin, where a difference (*p* < 0.01) between the novel and familiar objects was only observed within bin 1.

Literature utilizing rodents and sheep that focused on novelty and novelty approach behaviors may offer some explanation as to the spike in investigation after the first minute of introduction into the arena. Désiré et al. (2004, 2006) found that sudden introduction to novelty generated a startle response in sheep, delaying their investigations of the object. The authors suggest that sudden introduction of the object caused the sheep to see the object as a threat, rather than as neutral. Rodent studies found that mice are likely to investigate a novel object more when they also have free access to return to a familiar environment at any time during the trial (Misslin and Cigrang, 1986). The authors suggest that this is due to perceived control over the situation, subsequently providing more comfort in the situation. Taking these findings into consideration, it is possible that the sudden introduction into the arena, and as a result, to the novel object as well, may have caused a fear response in the pigs. The lack of ability to escape the environment may have further exacerbated the situation. How quickly pigs overcame the fear response would directly impact their investigative behaviors on an individual basis. As such, the peak in investigation of the novel object during the second minute of the trial may be explained by some pigs, but not all, beginning to overcome the initial fear response, allowing them to investigate. The speed at which pigs recovered from contextual fear may also account for the high variability observed in bin 2.

Binning with progressive minute inclusion (i.e., first minute, first 2 min, etc.) showed that both investigation time and number of investigations were significantly higher (*p* < 0.01) for the novel object compared to the familiar object across the progression of the test trial. The results of these analyses suggest that utilizing either just the first minute of data collection or any multi-minute bin maintains the differences observed in investigative behavior of the novel and familiar objects. That said, performing more granular analysis of the test trial (i.e., minute or multi-minute binning) is warranted to confirm the investigative differences. Most pig studies have utilized either a 5-min (Fil et al., 2021a; Fleming and Dilger, 2017; Martin et al., 2015) or 10-min (Fang et al., 2020; Kornum et al., 2007; Moustgaard et al., 2002; Søndergaard et al., 2012) testing duration. However, further investigation into trial length is warranted to confirm the findings from the above binning analyses. Future iterations should consider utilizing a 5- or 10-min test trial with the intention of performing more granular post-hoc analyses in order to pinpoint waning attention as a further measure of differences between treatment groups. This may provide further insight into differences caused by intervention.

### 3.5 How do the resultant data from the determined protocol compare to previous research?

The data analysis strategy used herein resulted in a dataset that focuses on controlling variance in RI values by applying data inclusion criteria focused on investigation time of and number of investigations with the novel object. The resultant data (*n* = 306) produce a mean RI value of 0.62 with an SD of 0.18, a CV of 30%, and an SE of 0.011. This is a reduction in RI variance from the full dataset (*n* = 367), which produced a CV of 40% and an SE of 0.024. Previous rodent studies have reported mean DI values of −0.1 to 0.1 with non-specific high levels of variation (Anderson et al., 2004; Reger et al., 2009). Pig studies also utilizing DI have reported mean values of −0.15 to 0.2, similarly mentioning high variation (Gifford et al., 2007; Kornum et al., 2007). Pig studies utilizing RI have reported mean values of 0.47 to 0.65 with SE values ranging from 0.032 to 0.077 (Fil et al., 2021a; Joung et al., 2020; Sutkus et al., 2022). Although the full dataset already produced a lower SE than the aforementioned studies, with the application of stricter data analysis techniques, variability in the RI value data was further reduced. However, it should be noted that different testing procedures were utilized among these studies, which introduces confounding factors to direct comparison.

These procedural discrepancies have also led to conflicting interpretations of results. Variations in the length of the delay period have led to a wide range of outcomes, causing difficulty in result comparisons (Fil et al., 2021a; Gifford et al., 2007; Joung et al., 2020; Kornum et al., 2007; Kouwenberg, 2008; Martin et al., 2015; Schmitt et al., 2019). These inconsistencies may also be caused by differences in testing arena design and location. For example, some iterations utilized a holding pen adjacent to the testing pen that allowed researchers to introduce pigs to the testing arena without researcher intervention (Kouwenberg, 2008; Martin et al., 2015; Schmitt et al., 2019), while others required hands-on tactics to introduce the pig into the arena (Fil et al., 2021a; Joung et al., 2020; Sutkus et al., 2022). Similarly, some testing arenas utilized bedding material (i.e., wood shavings or straw) (Moustgaard et al., 2002; Gifford et al., 2007) while others utilized raised, slatted flooring (Fleming and Dilger, 2017; Fleming et al., 2019; Fil et al., 2021a; Joung et al., 2020; Sutkus et al., 2022; Golden et al., 2024). While many studies performed the NOR paradigm in a separate room from where pigs were housed (Buddington et al., 2018; Fang et al., 2020; Kornum et al., 2007; Parois et al., 2021; Søndergaard et al., 2012), other variations performed the task in the same room (Gifford et al., 2007; Kaiser et al., 2021).

Procedural discrepancies also span to other aspects of the paradigm, such as the habituation and sample phases. For example, some iterations habituated animals to the testing apparatus for multiple days with multiple exposures per day (Kouwenberg, 2008; Martin et al., 2015; Parois et al., 2021; Schmitt et al., 2019), while others did little to no ( ≤ 1 exposure) habituation to the testing apparatus (Buddington et al., 2018; Fang et al., 2020; Kaiser et al., 2021). Techniques for habituating the animals to the familiar objects (i.e., sample phase) also differ in that some studies exposed pigs to the objects in the home pens/cages (Gifford, 2005; Gifford et al., 2007) while others exposed pigs to the familiar objects in the testing location (Fil et al., 2021a; Fleming et al., 2019; Golden et al., 2024).

## 4 Conclusion

In summary, this meta-analysis provides insight into the sources of variation in the data resulting from the novel object recognition task as used with young pigs. A total of 367 data points provided enough power to run various analyses on RI as well as exploratory behaviors. A histogram of RI values indicated a bimodal distribution skewed to the right. Furthermore, correlation analyses and regression models indicated that latency of investigations with the familiar object are not predictive of resultant RI values. However, future analyses should consider personality as a source of variability in relation to latency outcomes. Rather, cumulative investigation time and number of investigations with both the novel object and the familiar object, independently, most strongly predicted the resultant RI value. Subsequently, data inclusion criteria analyses focused exclusively on these four variables. Results indicated that requiring at least 5 s of investigation over at least 3 investigations with the novel object maintains 83% of the dataset while reducing the RI value variability. A closer look at the chosen model variables (novel object investigation time, number of novel object investigations, familiar object investigation time, number of familiar object investigations) indicated more overall investigation with the novel object across the entire trial as well as individual minutes for the first 4 min of the 5 min trial, with variability among the novel object exploratory behaviors being higher than exploratory behaviors of the familiar object. These results suggest 5-min test trials may be the appropriate amount of time for pigs to express inherent novelty preference before habituating to it. However, subsequent binning of the data is recommended to determine if/when attention to novelty in the environment begins waning. This meta-analysis focused exclusively on data obtained from the testing phase of NOR. Future analyses should consider a similar analytical breakdown as performed here of habituation and sample phase outcomes and their association with subsequent RI values. Similarly, future analyses should consider a similar analytical breakdown as performed here utilizing literature from multiple species.

## Data Availability

The raw data supporting the conclusions of this article will be made available by the authors, without undue reservation.

## Footnotes

1
http://loopbio.com/recording/
